# Factors influencing withdrawal of life-supporting treatment in cervical spinal cord injury: a large multicenter observational cohort study

**DOI:** 10.1186/s13054-023-04725-x

**Published:** 2023-11-18

**Authors:** Husain Shakil, Armaan K. Malhotra, Rachael H. Jaffe, Christopher W. Smith, Erin M. Harrington, Alick P. Wang, Eva Y. Yuan, Yingshi He, Karim Ladha, Duminda N. Wijeysundera, Avery B. Nathens, Jefferson R. Wilson, Christopher D. Witiw

**Affiliations:** 1 Division of Neurosurgery, Department of Surgery, University of Toronto, Toronto, ON Canada; 2 Li Ka Shing Knowledge Institute, St. Michael’s Hospital, Toronto, ON Canada; 3 Institute of Health Policy Management and Evaluation, University of Toronto, Toronto, ON Canada; 4 Department of Anesthesia, St. Michael’s Hospital, Toronto, ON Canada; 5 Department of Anesthesiology and Pain Medicine, University of Toronto, Toronto, ON Canada; 6 Division of Trauma Surgery, Sunnybrook Health Sciences Center, Toronto, ON Canada; 7 Division of Neurosurgery, Department of Surgery, University of Ottawa, Ottawa, ON Canada; 8 Division of Neurosurgery, Department of Surgery, St. Michael’s Hospital, 30 Bond Street, Toronto, ON M5B 1W8 Canada

**Keywords:** Traumatic spinal cord injury, Withdrawal of life-supporting treatment, End-of-life care, Equity of care, Interfacility variability

## Abstract

**Background:**

Traumatic spinal cord injury (SCI) leads to profound neurologic sequelae, and the provision of life-supporting treatment serves great importance among this patient population. The decision for withdrawal of life-supporting treatment (WLST) in complete traumatic SCI is complex with the lack of guidelines and limited understanding of practice patterns. We aimed to evaluate the individual and contextual factors associated with the decision for WLST and assess between-center differences in practice patterns across North American trauma centers for patients with complete cervical SCI.

**Methods:**

This retrospective multicenter observational cohort study utilized data derived from the American College of Surgeons Trauma Quality Improvement Program database between 2017 and 2020. The study included adult patients (> 16 years) with complete cervical SCI. We constructed a multilevel mixed effect logistic regression model to adjust for patient, injury and hospital factors influencing WLST. Factors associated with WLST were estimated through odds ratios with 95% confidence intervals. Hospital variability was characterized using the median odds ratio. Unexplained residual variability was assessed through the proportional change in variation between models.

**Results:**

We identified 5070 patients with complete cervical SCI treated across 477 hospitals, of which 960 (18.9%) had WLST. Patient-level factors associated with significantly increased likelihood of WLST were advanced age, male sex, white race, prior dementia, low presenting Glasgow Coma Scale score, having a pre-hospital cardiac arrest, SCI level of C3 or above, and concurrent severe injury to the head or thorax. Patient-level factors associated with significantly decreased likelihood of WLST included being racially Black or Asian. There was significant variability across hospitals in the likelihood for WLST while accounting for case-mix, hospital size, and teaching status (MOR 1.51 95% CI 1.22–1.75).

**Conclusions:**

A notable proportion of patients with complete cervical SCI undergo WLST during their in-hospital admission. We have highlighted several factors associated with this decision and identified considerable variability between hospitals. Further work to standardize WLST guidelines may improve equity of care provided to this patient population.

**Supplementary Information:**

The online version contains supplementary material available at 10.1186/s13054-023-04725-x.

## Background

Traumatic spinal cord injury (SCI) has been estimated to affect 1.3 million North Americans [1]. Such an injury can be a devastating event with profound consequences for patients, caregivers, and the healthcare system [2, 3]. In the setting of complete cervical SCI, patients suffer from neurologic sequelae including quadriplegia, loss of sensation and potential loss of respiratory control, which can affect a patient’s ability to move, breathe, and perform activities of daily living. The provision of life-supporting therapy is therefore often a central component of medical care in this patient population. Some patients or caregivers choose to explore the option of withdrawal of life-supporting treatment (WLST). The decision for WLST is a complex process that requires careful consideration of patient values, clinical status, prognosis, support networks, and quality of life. The physician’s role is to help guide decision makers through this challenging terrain by providing objective evaluations of different available treatment pathways. Despite this, there are limited guidelines around how to navigate this complex decision for patients with complete cervical SCI.

To date, there are no multicenter studies describing factors associated with WLST in the SCI population. Current literature on this topic is scarce, with most studies being limited to case reports or series [4–6]. An understanding of practice patterns with respect to WLST across health care centers treating patients with this condition is equally lacking. These knowledge gaps represent key areas to be addressed to ensure high-quality care for patients suffering from traumatic SCI. The aim of this study was to assess contemporary trends in the practice of WLST and identify individual and contextual factors associated with the decision for WLST among patients with complete cervical SCI. Secondary aims of this study were to assess variation in practice patterns across North American trauma centers with respect to the decision for WLST.

## Methods

### Study design and data source

We conducted a retrospective observational cohort study. All data was derived from the American College of Surgeons (ACS) Trauma Quality Improvement Program (TQIP) database from 2017 to 2020 [7]. Nearly 900 ACS- and state-verified level I, II, and III trauma centers across North America contribute to the TQIP database for the purpose of quality improvement. Over 300 variables are collected for every trauma admission each year, including patient demographic and injury characteristics, comorbidities, processes of care, and outcomes. It includes all patients from participating centers with at least one severe injury (Abbreviated Injury Scale (AIS) ≥ 3 in at least one body region). Data reliability and quality are maintained through training of data abstractors and inter-rater reliability audits of contributing centers.

### Participants

**Fig. 1:**
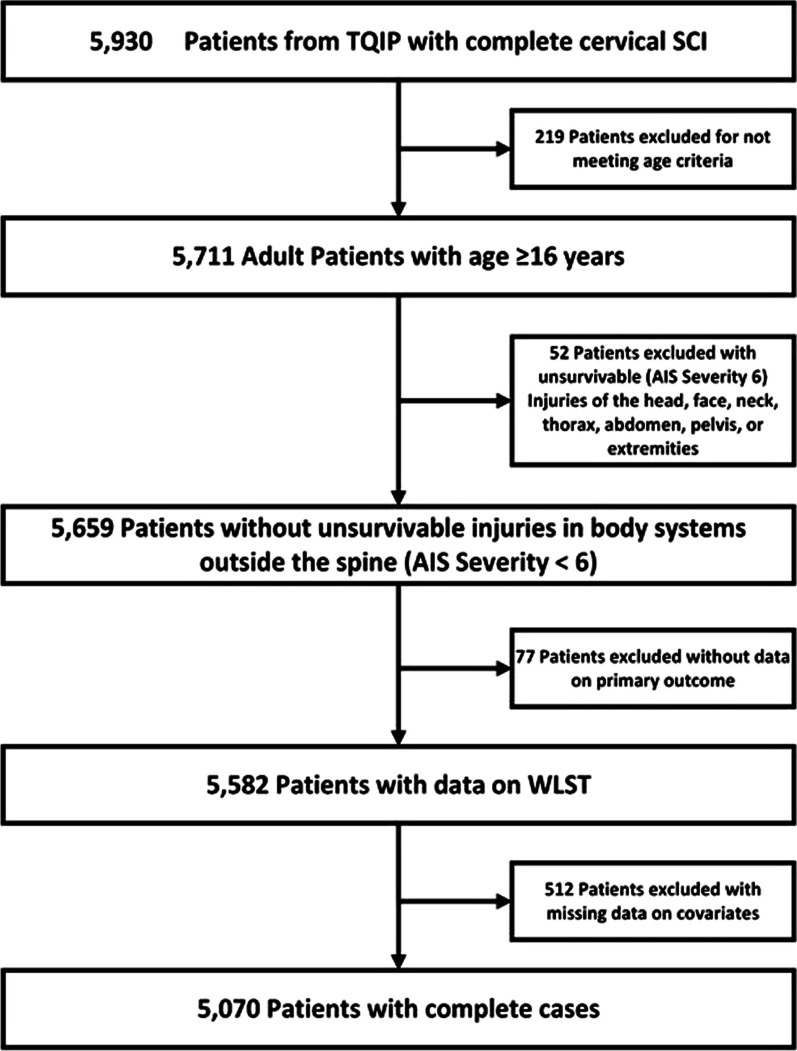
Study cohort creation diagram in accordance with the RECORD statement. *Abbreviations* TQIP, Trauma Quality Improvement Program; SCI, spinal cord injury; AIS, Abbreviated Injury Scale; WLST, withdrawal of life-supporting treatment

The study cohort was created and reported in accordance with the Reporting of studies Conducted using Observational Routinely collected health Data (RECORD) statement (Fig. 1) [8]. Adult patients (≥ 16 years) with a diagnosis of an acute traumatic American Spinal Injury Association Grade A cervical SCI were included [9]. This definition represents a complete sensory and motor injury and was identified using AIS codes (Additional file 1). Patients identified with complete cervical SCI and additional non-spine injuries with an AIS score of 6 were also excluded, as these are considered non-survivable injuries [10]. Patients without data on WLST were excluded. Moreover, patients with missing data on demographic, injury, and hospital variables used for regression were also excluded. Additionally, patients with pre-existing advanced directive limiting care are excluded from TQIP.

### Variables

Several patient and hospital variables were selected from the TQIP database according to clinical relevance. Patient demographic data included age, sex, race, and medical insurance type. Age was treated as a continuous variable; sex was dichotomized into male and female; and race was categorized as racially Black, White, Asian, and other. These categories were based on coding available in TQIP, and to ensure a minimum of 5 WLST observations per category as recommended by Harrell [11]. Insurance type was categorized as Medicaid, Medicare, private, self-pay, and other. These categories were chosen based on the data coding in TQIP and to ensure a minimum of 5 WLST observations per category to adequately adjust for each variable level in the regression model. We also collected data on comorbidities likely to influence a decision for WLST, such as whether a patient was functionally dependent prior to injury, or had a history of stroke, dementia, disseminated cancer, or chronic renal failure. Each of these data were available as binary variables in TQIP. Data on the characteristics of the hospital presentation and traumatic injury were also collected. This included the presenting Glasgow Coma Scale (GCS); presence of shock (defined as initial Emergency Department blood pressure < 90 mmHg), whether a patient sustained a pre-hospital cardiac arrest; mechanism of injury; and AIS body region scores including head, face, neck, thorax, abdomen, and the upper and lower extremities. We also captured data on the anatomic level of the SCI, specifically if the injury was high cervical, defined to be C3 and above, or lower cervical defined to be C4 and below. The patient’s GCS was categorized as follows: GCS15, GCS13–14, GCS 9–12, GCS 3–8, consistent with categories corresponding to severity of traumatic brain injury [21]. Mechanism of injury was categorized as blunt or penetrating. This type of categorization has been used in prior studies [12–14]. AIS body region scores were dichotomized into 1–2 (minor–moderate injuries) and ≥ 3 (serious–critical injuries). This grouping has also been used in prior studies on SCI [13, 14]. Hospital characteristics including the size, and teaching status were also captured. Hospital size was categorized as ≤ 200 beds, 201–400 beds, 400–600, and > 600 beds. Hospital teaching status was categorized as university hospital, community hospital, and non-teaching hospital. We also were able to link each trauma admission to a unique hospital facility key, to cluster patients according to the hospital at which they were treated.

### Outcome

The primary outcome was WLST occurring from the time of hospital arrival to discharge, transfer, or death. TQIP defines WLST to be the decision to either limit escalation, remove, or withhold further life-supporting intervention, limited to ventilator support, dialysis, other forms of renal support, medications to support blood pressure or cardiac function, or a specific surgical, interventional, or radiological procedure. Decisions for WLST were required to be documented in the medical record through either a physician order, progress note, case manager note, social services note, nursing note, nursing flowsheet, or discharge summary. A Do Not Resuscitate status was not a requirement nor seen as equivalent to WLST. Furthermore, TQIP excludes the discontinuation of cardiopulmonary resuscitation from the outcome definition. WLST was coded as a binary variable within TQIP.

### Statistical analyses

All statistical analyses were performed using R version 4.2.1 with an a priori specified significance level of *p* = 0.05 for two-tailed tests. Descriptive statistics were reported as mean, standard deviation, minimum, median, and maximum for continuous variables and count with proportions for categorical variables. Covariate balance across the cohort was assessed by comparing the absolute standardized difference between patients with and without WLST, with a pre-specified threshold of 0.1 representing a significant difference between groups [15].

### Multilevel logistic regression

A multilevel mixed effects logistic regression model was constructed with WLST as the primary outcome. We followed multilevel logistic regression best practices outlined by Austin and Merlo [16, 17]. Fixed effect model covariates included age, sex, race, insurance type, functionally dependent status, history of stroke, history of dementia, history of disseminated cancer, history of chronic renal failure, presenting GCS, presence of shock, presence of pre-hospital cardiac arrest, mechanism of injury, head, face, neck, thorax, and abdomen AIS scores, spinal cord level of injury, hospital size, hospital teaching status, and year of injury. Age was modeled as a nonlinear function using restricted cubic splines with 4 knots located at the 5th, 35th, 65th, and 95th quantiles (Additional files 2, 3, 4 and 5).

To investigate differences in case-mix and unmeasured variability between hospitals, we modeled hierarchical clustering of data at the hospital level through random intercepts. We treated hospital specific facility identifiers as a random effect in our multilevel logistic regression model. Effect sizes for fixed effects were reported as odds ratios (OR) with 95% confidence intervals (CI). For age, due to the use of non-linear splines, we reported the OR associated with a change in age from 20 to 30 years, and 60–70 years. Point estimates were given using the *predict()* function, and 95% confidence intervals were computed using a bootstrap with 100 iterations. We computed *p*-values with Wald’s test [11] for all fixed effects other than age. For age, due to the use of splines, we reported the *p*-value associated with a likelihood ratio test when comparing a full model, with a nested model not including age as a covariate [11]. Between hospital practice pattern variation with respect to WLST was quantified through the median odds ratio (MOR). A 95% confidence interval was computed for the MOR using a bootstrap with 100 iterations [16, 17]. The MOR represents the odds of WLST for a patient admitted to a hospital relative to the same patient admitted to another randomly selected hospital. It provides a measure of between-cluster variation in the odds of WLST, providing an estimate of the contextual effect for the decision for WLST. Multilevel model analyses have been well described in a series of prior papers by Merlo and Austin [16–20]. In addition, we computed the proportional change in variance (PCV) attributable to measured patient-level and hospital-level covariates by comparing the difference in individual-level variance between a ‘null model’ including only the hospital random effect, with a ‘full model’ including both the full complement of fixed along with the hospital random effects. We also computed the MOR for the null model for comparison.

A sensitivity analysis was done by refitting the full model after excluding all patients with a presenting GCS of 3-8 (Additional file 6). This was done to further address any confounding related to a concurrent severe traumatic brain injury, as this has been shown to influence the decision for WLST [21].

Missing data were analyzed and found to be missing at random, with less than 5% missing data per variable and less than 10% across all variables (Additional files 7 and 8). We conducted a complete cases analysis and did not impute missing data for the model.

## Results

Within the 2017–2020 TQIP database, we identified 5070 patients treated across 477 hospitals that that met our study criteria (Fig. 1). Baseline characteristics of the cohort are summarized in Table 1. Mean age for the cohort was 49.6 years. Most patients were male (80.2%) and racially white (63.7%). The highest proportion of patients had private insurance as their payer (39.5%). Within the cohort, 960 (18.9%) patients had WLST across 329 of 477 hospitals (69.0%). Assessment of covariate balance demonstrated significant differences between patients with and without WLST with respect to age, race, insurance type, functional dependence, dementia, presenting GCS, shock, pre-hospital cardiac arrest, mechanism of injury, SCI level, severe concurrent head injuries, and severe concurrent thoracic injuries (Table 1).

**Table 1:** Baseline characteristics of study cohort with comparisons based on decision for withdrawal of life-supporting treatment

	Total cohort *n* = 5070	No WLST *n* = 4110 (81.1%)	WLST *n* = 960 (18.9%)	Absolute standardized difference
Patient-level characteristics				
Age (years)				0.72^a^
Mean (SD)	49.6 (20.1)	46.4 (19.2)	62.9 (18.0)	
Median [Min, Max]	52.0 [16.0, 89.0]	47.0 [16.0, 89.0]	67.0 [16.0, 89.0]	
Sex—*n* (%)				0.01
Female	1005 (19.8%)	819 (19.9%)	186 (19.4%)	
Male	4065 (80.2%)	3291 (80.1%)	774 (80.6%)	
Race—*n* (%)				0.45^a^
White	3228 (63.7%)	2462 (59.9%)	766 (79.8%)	
Black	1267 (25.0%)	1142 (27.8%)	125 (13.0%)	
Asian	135 (2.7%)	119 (2.9%)	16 (1.7%)	
Other	440 (8.7%)	387 (9.4%)	53 (5.5%)	
Insurance type—*n* (%)				0.62^a^
Private/Commercial Insurance	2001 (39.5%)	1708 (41.6%)	293 (30.5%)	
Medicaid	1090 (21.5%)	987 (24.0%)	103 (10.7%)	
Medicare	1154 (22.8%)	735 (17.9%)	419 (43.6%)	
Self-pay	519 (10.2%)	421 (10.2%)	98 (10.2%)	
Other	306 (6.0%)	259 (6.3%)	47 (4.9%)	
Comorbidities—*n* (%)				
Functionally dependent	180 (3.6%)	119 (2.9%)	61 (6.4%)	0.17^a^
Prior stroke	84 (1.7%)	58 (1.4%)	26 (2.7%)	0.09
Dementia	85 (1.7%)	39 (1.0%)	46 (4.8%)	0.23^a^
Disseminated cancer	33 (0.7%)	21 (0.5%)	12 (1.3%)	0.08
Chronic renal failure	51 (1.0%)	35 (0.9%)	16 (1.7%)	0.07
Presenting Glasgow Coma Scale—*n* (%)				0.70^a^
15	2504 (49.4%)	2233 (54.3%)	271 (28.2%)	
13–14	651 (12.8%)	555 (13.5%)	96 (10.0%)	
9–12	428 (8.4%)	363 (8.8%)	65 (6.8%)	
3–8	1487 (29.3%)	959 (23.3%)	528 (55.0%)	
Shock in emergency department—*n* (%)	294 (5.8%)	211 (5.1%)	83 (8.6%)	0.14^a^
Pre-hospital cardiac arrest—*n* (%)	562 (11.1%)	288 (7.0%)	274 (28.5%)	0.59^a^
Mechanism of injury—*n* (%)				0.11^a^
Blunt	4590 (90.5%)	3697 (90.0%)	893 (93.0%)	
Penetrating	480 (9.5%)	413 (10.0%)	67 (7.0%)	
Spinal cord level of injury—*n* (%)				0.59^a^
C4 and below	3933 (77.6%)	3392 (82.5%)	541 (56.4%)	
C3 and above	1137 (22.4%)	718 (17.5%)	419 (43.6%)	
Severe non-spinal injuries (AIS ≥ 3)—*n* (%)				
Head	1004 (19.8%)	710 (17.3%)	294 (30.6%)	0.32^a^
Face	31 (0.6%)	22 (0.5%)	9 (0.9%)	0.05
Neck	648 (12.8%)	511 (12.4%)	137 (14.3%)	0.05
Thorax	986 (19.4%)	735 (17.9%)	251 (26.1%)	0.20^a^
Abdomen	185 (3.6%)	146 (3.6%)	39 (4.1%)	0.03
Upper extremities	42 (0.8%)	34 (0.8%)	8 (0.8%)	< 0.01
Lower extremities	271 (5.3%)	200 (4.9%)	71 (7.4%)	0.11^a^
Hospital-level characteristics				
Hospital size (Beds)—*n* (%)				0.11^a^
< = 200	202 (4.0%)	165 (4.0%)	37 (3.9%)	
201–400	1214 (23.9%)	969 (23.6%)	245 (25.5%)	
401–600	1553 (30.6%)	1232 (30.0%)	321 (33.4%)	
> 600	2101 (41.4%)	1744 (42.4%)	357 (37.2%)	
Hospital teaching status—*n* (%)				0.05
University	2835 (55.9%)	2316 (56.4%)	519 (54.1%)	
Non-teaching	521 (10.3%)	424 (10.3%)	97 (10.1%)	
Community	1714 (33.8%)	1370 (33.3%)	344 (35.8%)	
Year of injury—*n* (%)				0.08
2017	1255 (24.8%)	996 (24.2%)	259 (27.0%)	
2018	1249 (24.6%)	1009 (24.5%)	240 (25.0%)	
2019	1230 (24.3%)	998 (24.3%)	232 (24.2%)	
2020	1336 (26.4%)	1107 (26.9%)	229 (23.9%)	
Number of treating hospitals	477 (100%)	469 (98.3%)	329 (69.0%)	-

^a^Statistically significant

*WLST* Withdrawal of life-supporting treatment, *SD* Standard deviation, AIS, Abbreviated Injury Scale

### Patient and hospital factors associated with withdrawal of life-supporting treatment

**Fig. 2:**
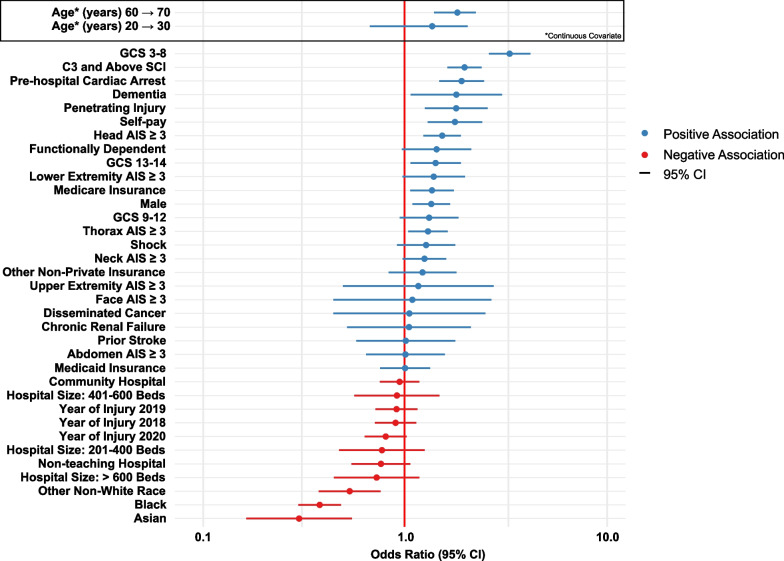
Forest plot of adjusted odds ratios for patient-level and hospital-level fixed effect predictors of WLST. Adjusted odds ratios were estimated using multilevel logistic regression, with point estimates ranked according to effect size. Predictors with point estimates corresponding to a positive association with WLST are shown in blue, while negative associations are shown in red. *Abbreviations*: AIS, Abbreviated Injury Scale; WLST, withdrawal of life-supporting treatment; SCI, spinal cord injury; GCS, Glasgow Coma Scale; CI, confidence interval

Results for the multilevel logistic regression model for WLST with respect to fixed effects are summarized in Table 2 and Fig. 2. The likelihood for WLST was found to be significantly associated with age with a non-linear relationship. The odds ratio of WLST associated with a 10-year increase of age, from 60 to 70 years was larger than that associated with an increase from 20 to 30 years. Other patient factors found to significantly increase likelihood of WLST, after adjustment, included being male, having Medicare, self-pay, and a prior history of dementia. Conversely, being Black or Asian significantly decreased the likelihood of WLST.

**Table 2:** Summary of patient- and hospital-level fixed effect predictors associated with withdrawal of life-supporting treatment

Predictors	Odds ratios	95% CI	*p*-value
Patient-level characteristics			
Age (years)			< 0.001^a,b^
Change from 20 to 30 years	1.37	0.67–2.06	
Change from 60 to 70 years	1.83	1.40–2.26	
Sex—reference: female			
Male	1.36	1.09–1.68	0.006^a^
Race—reference: white			
Black	0.38	0.29–0.48	< 0.001^a^
Asian	0.30	0.16–0.54	< 0.001^a^
Other	0.53	0.37–0.76	< 0.001^a^
Insurance type—reference: private/commercial			
Medicaid	1.00	0.75–1.34	0.983
Medicare	1.37	1.06–1.76	0.014^a^
Self-Pay	1.78	1.30–2.44	< 0.001^a^
Other	1.23	0.83–1.81	0.306
Comorbidities			
Functionally dependent	1.44	0.97–2.15	0.072
Prior Stroke	1.01	0.57–1.79	0.969
Dementia	1.81	1.07–3.06	0.027^a^
Disseminated Cancer	1.05	0.44–2.52	0.907
Chronic Renal Failure	1.05	0.52–2.14	0.894
Presenting Glasgow Coma Scale—reference: 15			
3–14	1.42	1.07–1.90	0.016^a^
9–12	1.32	0.94–1.85	0.105
3–8	3.34	2.63–4.24	< 0.001^a^
Shock	1.28	0.91–1.78	0.151
Pre-hospital cardiac arrest	1.92	1.49–2.48	< 0.001^a^
Mechanism of injury—reference: blunt			
Penetrating	1.80	1.26–2.59	0.001
Spinal cord level of injury—reference: C4 and below			
C3 and above	1.99	1.63–2.42	< 0.001
Severe non-spinal injury—reference: Body system AIS < 3			
Head AIS ≥ 3	1.54	1.24–1.91	< 0.001^a^
Face AIS ≥ 3	1.09	0.44–2.70	0.852
Neck AIS ≥ 3	1.25	0.98–1.61	0.078
Thorax AIS ≥ 3	1.31	1.04–1.64	0.021^a^
Abdomen AIS ≥ 3	1.01	0.64–1.58	0.973
Upper extremity AIS ≥ 3	1.17	0.49–2.78	0.725
Lower extremity AIS ≥ 3	1.39	0.97–2.00	0.070
Hospital-level characteristics			
Hospital size (Beds)—reference: ≤ 200			
201–400	0.77	0.47–1.26	0.294
401–600	0.91	0.56–1.49	0.716
> 600	0.72	0.44–1.18	0.195
Hospital teaching status—reference: university			
Non-teaching	0.76	0.54–1.07	0.111
Community	0.94	0.75–1.18	0.602
Year of injury—reference: 2017			
2018	0.90	0.71–1.14	0.381
2019	0.91	0.71–1.16	0.439
2020	0.80	0.63–1.02	0.074

^a^Statistically significant

^b^Likelihood ratio test

*CI* Confidence interval, *AIS* Abbreviated injury scale

Injury factors associated with significantly increased likelihood for decision for WLST included presenting to hospital in a comatose state with GCS 3–8 (OR 3.29 95% CI 2.60–4.18), with a pre-hospital cardiac arrest (OR 1.92 95% CI 1.48–2.48), or with a penetrating injury (OR 1.79 95% CI 1.25–2.56). An injury level of C3 and above was associated with twice the odds for WLST compared to an injury of C4 and below (OR 2.00 95% CI 1.65–2.44). With respect to polytrauma patients, having a concurrent severe head injury (OR 1.55 95% CI 1.25–1.91) or thoracic injury (OR 1.29 95% CI 1.03–1.62) was associated with significantly increased likelihood for WLST.

A sensitivity analysis of results after excluding patients with GCS 3–8 was consistent with the primary analysis and found that age, male sex, self-pay, lower GCS, concurrent severe thoracic injury, and a higher SCI level were significantly associated with a higher likelihood of WLST; and being black was significantly associated with a lower likelihood of WLST. Complete results from the sensitivity analysis can be found in Additional file 6.

### Between hospital variation in practice patterns of withdrawal of life-supporting treatment

Results of the hospital-level clustering analysis of WLST are provided in Table 3. The average intercept and hospital-level variance of the null model on the log-odds scale were − 1.49 and 0.18, respectively. When computing equivalent values for the full model with adjustment for covariates, the average intercept and hospital-level variation were − 4.07 and 0.17, respectively. The MOR of the full model was 1.48 (95% CI 1.22–1.75), highlighting a significant variation in practice patterns between hospitals. The PCV in the null model after inclusion of patient and hospital covariates was 5.8%, suggesting 94.2% of the variation in hospital-level practices for WLST is unmeasured by the covariates included in the model.

**Table 3:** Random effects at the hospital level, clustering, and model fit for multilevel logistic regression models

	Null model	Multilevel model with adjustment for patient and hospital covariates
Average intercept (Log-odds scale)	− 1.50	− 4.16
Hospital-level variance (Log-odds scale)	0.18	0.17
Median odds ratio—MOR (95% CI)	1.50 (1.31–1.69)	1.48 (1.22–1.75)
Proportional change in variance—PCV	Reference	5.8%

## Discussion

In this multicenter retrospective cohort study, we identified factors associated with WLST and investigated variation in practice patterns for WLST among patients with complete cervical SCI. In our study, 18.9% of patients had WLST. Patient demographic factors found to be significantly associated with the decision for WLST included age, sex, race, type of medical insurance, and prior history of dementia. Injury factors found to be significantly associated with WLST included low presenting GCS, presence of a pre-hospital cardiac arrest, penetrating injuries, a C3 and above SCI, and a concurrent severe head or thorax injury. We noted significant variation in practice patterns, with a 95% CI MOR of 1.22–1.75 for the adjusted model, suggesting a significant difference in the odds ratio for WLST in a standard patient from any two hospitals across the study.

Despite its relevance, there have been surprisingly limited prior studies investigating WLST in patients with acute SCI. Osterthun et al. conducted a retrospective study on end-of-life decisions among patients treated with acute SCI across hospital in the Netherlands in 2010. Of the 185 SCI patients treated that year, they noted 19 (10.3%) were reported to have WLST [22]. We noted 18.9% of cases across our study population had WLST. However, our study was focused on complete cervical SCI, which represents a severe form of SCI, and a subset of the heterogenous population of patients with acute SCI. Consideration of WLST is likely more relevant in this patient population as they suffer specifically from quadriplegia and often require respiratory support. This difference likely accounts for the higher rate noted in our study. To our knowledge, all other studies reporting cases of WLST in acute SCI are limited to case reports and case series [4–6].

Other studies have assessed factors associated with WLST and practice patterns of WLST in the setting of other traumatic injuries. For patients with severe traumatic brain injury, Williamson et al. noted a WLST rate of 20.7% across the cohort of injured patients [21]. Similar to our study, they noted that race and medical insurance type, and prior history of dementia were significantly associated with decision for WLST in the setting of severe traumatic injury. In their study, however, they did note a significant association between hospital teaching status and the decision for WLST. However, in their study they did not account for hospital-level clustering, which may narrow the confidence intervals around their estimates [16–20]. A prospective cohort study conducted by Cooper et al. looked at variation in practice patterns for withdrawal of care orders for patients with any severe traumatic injury (AIS ≥ 3) across various trauma and non-trauma centers distributed among 12 states [23]. They identified 14,190 patients, of which 618 (4.4%) of patients had a withdrawal of care order. This suggests that patients with complete cervical SCI reflect a subset of the trauma patient population where WLST is more common. Similar to our study, Cooper et al. also noted that age, race, comorbidities, and lower presenting GCS were associated with a withdrawal of care order for trauma patients in general.

In our study we found race and medical insurance type were significantly associated with WLST. Black and Asian patients were less likely to have WLST, and patients with Medicaid or Medicare more likely to have WLST. This highlights potential equity issues within decision-making for WLST among patients with complete cervical SCI. Racial disparities in trauma care have previously been identified. Duong et al. investigated mortality among trauma patients in the USA admitted between 2010 and 2016, and found black patients had an OR of mortality as high as 1.30 when compared to white patients [24]. Furthermore, differences in WLST associated with patient medical insurance type may reflect significant equity issues related to cost of treatment and long-term care. Evidence for disparities in trauma care based on insurance status has been found. A single-center study by de Angelis et al. on trauma patients admitted between 2016 and 2018 found uninsured patients more likely to have a shorter hospital length of stay (incidence rate ratio 0.34, 95% CI 0.24–0.51) [25]. Further research addressing these socioeconomic disparities is crucial to ensure all patients receive equitable, high-quality care.

Limitations of our study are largely imparted by the source of data available. TQIP is a rigorously maintained database, with regular audits and training of data abstractors, however the presence of possible misclassification in data elements is always present in secondary data sources. However, there is no strong reason to suspect differential misclassification among patients with and without WLST. We noted less than 5% missing data among variables relevant to our study; as such, we do not suspect this to severely bias the inference from our study. Furthermore, our analysis of predictors was limited to variables available in the database. We did not have any data on patient religious affiliation, attending physician religious affiliation, and family social dynamics, which would be important for better understanding of decisions around WLST among our patient population [26]. Generalizability of findings is another limitation. TQIP derives data from trauma centers with specific focus on quality of care. As such, we have likely underestimated the variation in practice patterns with respect to WLST that would be found across all hospitals in North America.

A strength of this study is the large sample size of 5070 patients, with 960 observations of WLST across 477 North American institutions over 4 years. Therefore, we me had sufficient observations to regress on the 39 model coefficients included in the model. In addition, our use of a multilevel mixed effect model allowed for the estimation of effect sizes, while adjusting for patient- and hospital-level confounders and data clustering.

## Conclusions

An understanding of practice patterns for WLST are relevant to providing quality care to patients with complete cervical SCI. In the current study, we have found that 18.9% of patients with complete cervical SCI ultimately had WLST. Both patient demographic and injury-related factors are associated with this decision, with notable practice variation across North American institutions. More studies are needed investigating the source of variability across hospitals, which is important for hospital policy makers aiming to maintain quality of care. Overall, our study points to a need for standardized WLST guidelines to improve quality of care for patients with complete cervical SCI.

### Supplementary Information

**Additional file 1**. Acute Injury Severity codes used to identify complete traumatic spinal cord injury patients within the Trauma Quality Improvement Program.

**Additional file 2**. Modelling Age as non-linear covariate.

**Additional file 3**. Results from analysis comparing a logistic regression model for age as a linear term, and non-linear terms. The table demonstrates the results from an analysis of variance of model terms from a non-linear model fit with age fit with restricted cubic splines with 4 knots. The likelihood ratio test^a^ compares this model, with a nested model not including the non-linear terms. Abbreviations: GCS, Glasgow Coma Scale; AIS, Abbreviated Injury Scale

**Additional file 4**. Partial residual plots of a logistic regression model fit with age as a linear predictor demonstrates a residual trend.

**Additional file 5**. Results from a full model with all covariates with age fit using restricted cubic splines demonstrates a non-linear relationship between the log-odds of withdrawal of life-supporting testament and age. Plots are adjusted to: Male sex; white race; private insurance; absence of functionally dependent status, prior stroke, dementia, disseminated cancer, chronic renal failure, shock, pre-hospital cardiac arrest; Glasgow Coma Scale score 15; Blunt Traumas; Abbreviated Injury Scale 1-2 score for head, face, neck, thorax, abdomen, upper extremity, and lower extremity; C4 and below spine injury; hospital bed size ≥ 600, university hospitals, injury year 2020. The grey shaded region represents a 95% confidence interval. Abbreviations: WLST, withdrawal of life-supporting treatment.

**Additional file 6**. Results from sensitivity analysis excluding patients with presenting Glasgow Coma Scale between 3-8. The table provides estimated odds ratios for fixed effect predictors with a multilevel logistic regression model for withdrawal of life-supporting treatment. Abbreviations: CI, confidence interval; AIS, Abbreviated Injury Scale.

**Additional file 7**. Analysis of missing data demonstrates that covariates are not missing completely at random based on Little’s Test^a^. Proportions of missing data by covariate and separated by complete cases and those with at least one element of missing data. Absolute standardized differences are tabulated. An absolute standardized difference >0.1 was prespecified to represent a meaningful difference between the group of patients with missing data and the group with complete cases. On inspection, there is no systematic trend in missing variables, suggesting they are likely missing at random. Abbreviations: GCS, Glasgow Coma Scale; AIS, Abbreviated Injury Scale.

**Additional file 8**. Percentage of missing data by variable demonstrates that less than 5% data are missing per covariate. Abbreviations: GCS, Glasgow Coma Scale; AIS, Abbreviated Injury Scale.

## Data Availability

The data collected for this study will not made available to others because restrictions apply to the availability of this data, which were used under license for the current study. The data that support the findings of this study is owned by the American College of Surgeons (ACS) Trauma Quality Improvement Program (TQIP) and is publicly available upon request. The corresponding author of this paper can be contacted for guidance in requesting access to this data.

## Abbreviations

ACS: American College of Surgeons
AIS: Abbreviated Injury Scale
CI: Confidence interval
GCS: Glasgow Coma Scale
MOR: Median odds ratio
OR: Odds ratio
PCV: Proportional change in variance
RECORD: Reporting of studies Conducted using Observational Routinely collected health Data
SCI: Spinal cord injury
TQIP: Trauma Quality Improvement Program
WLST: Withdrawal of life-supporting treatment

## References

[1] Ahuja CS, Martin AR, Fehlings M (2016). Recent advances in managing a spinal cord injury secondary to trauma. F1000Res.

[2] Witiw CD, Fehlings MG (2015). Acute spinal cord injury. J Spin Disord Tech.

[3] Ahuja CS, Wilson JR, Nori S, Kotter MRN, Druschel C, Curt A (2017). Traumatic spinal cord injury. Nat Rev Dis Prim.

[4] Patterson DR, Miller-Perrin C, McCormick TR, Hudson LD (1993). When life support is questioned early in the care of patients with cervical-level quadriplegia. N Engl J Med.

[5] Taub AL, Keune JD, Kodner IJ, Schwarze ML (2014). Respecting autonomy in the setting of acute traumatic quadriplegia. Surgery.

[6] Field HL (2008). A patient with acute traumatic quadriplegia who requested a DNR order. Psychosomatics.

[7] Nathens AB, Cryer HG, Fildes J (2012). The American college of surgeons trauma quality improvement program. Surg Clin North Am.

[8] Langan SM, Schmidt SA, Wing K, Ehrenstein V, Nicholls SG, Filion KB (2018). The reporting of studies conducted using observational routinely collected health data statement for pharmacoepidemiology (RECORD-PE). BMJ.

[9] Roberts TT, Leonard GR, Cepela DJ (2017). Classifications in brief: American spinal injury association (ASIA) impairment scale. Clin Orthop Relat Res.

[10] Greenspan L, McLellan BA, Greig H (1985). Abbreviated injury scale and injury severity score: a scoring chart. J Trauma.

[11] Harrell FE (2015). Regression modeling strategies.

[12] Shakil H, Jaja BNR, Zhang PF, Jaffe RH, Malhotra AK, Harrington EM (2023). Assessment of the incremental prognostic value from the modified frailty index-5 in complete traumatic cervical spinal cord injury. Sci Rep.

[13] Balas M, Guttman MP, Badhiwala JH, Lebovic G, Nathens AB, Da Costa L (2022). Earlier surgery reduces complications in acute traumatic thoracolumbar spinal cord injury: analysis of a multi-center cohort of 4108 patients. J Neurotrauma.

[14] Badhiwala JH, Lebovic G, Balas M, da Costa L, Nathens AB, Fehlings MG (2021). Variability in time to surgery for patients with acute thoracolumbar spinal cord injuries. Sci Rep.

[15] Austin PC (2009). Balance diagnostics for comparing the distribution of baseline covariates between treatment groups in propensity-score matched samples. Stat Med.

[16] Merlo J, Chaix B, Ohlsson H, Beckman A, Johnell K, Hjerpe P (2006). A brief conceptual tutorial of multilevel analysis in social epidemiology: using measures of clustering in multilevel logistic regression to investigate contextual phenomena. J Epidemiol Commun Health.

[17] Austin PC, Merlo J (2017). Intermediate and advanced topics in multilevel logistic regression analysis. Stat Med.

[18] Merlo J, Chaix B, Yang M, Lynch J, Råstam L (2005). A brief conceptual tutorial on multilevel analysis in social epidemiology: interpreting neighbourhood differences and the effect of neighbourhood characteristics on individual health. J Epidemiol Community Health.

[19] Merlo J, Yang M, Chaix B, Lynch J, Råstam L (1978). A brief conceptual tutorial on multilevel analysis in social epidemiology: investigating contextual phenomena in different groups of people. J Epidemiol Community Health.

[20] Merlo J, Chaix B, Yang M, Lynch J, Råstam L (1978). A brief conceptual tutorial of multilevel analysis in social epidemiology: linking the statistical concept of clustering to the idea of contextual phenomenon. J Epidemiol Community Health.

[21] Williamson T, Ryser MD, Ubel PA, Abdelgadir J, Spears CA, Liu B (2020). Withdrawal of life-supporting treatment in severe traumatic brain injury. JAMA Surg.

[22] Osterthun R, Van Asbeck FWA, Nijendijk JHB, Post MWM (2016). In-hospital end-of-life decisions after new traumatic spinal cord injury in the Netherlands. Spinal Cord.

[23] Cooper Z, Rivara FP, Wang J, MacKenzie EJ, Jurkovich GJ (2009). Withdrawal of life sustaining therapy in injured patients: variations between trauma centers and non-trauma centers. J Trauma.

[24] Duong WQ, Grigorian A, Farzaneh C, Nahmias J, Chin T, Schubl S (2021). Racial and sex disparities in trauma outcomes based on geographical region. Am Surg.

[25] de Angelis P, Kaufman EJ, Barie PS, Leahy NE, Winchell RJ, Narayan M (2022). Disparities in insurance status are associated with outcomes but not timing of trauma care. J Surg Res.

[26] Garg A, Soto AL, Knies AK, Kolenikov S, Schalk M, Hammer H (2021). Predictors of surrogate decision makers selecting life-sustaining therapy for severe acute brain injury patients: an analysis of US population survey data. Neurocrit Care.

