# Supplementary material for: Factors influencing withdrawal of life-supporting treatment in cervical spinal cord injury: a large multicenter observational cohort study
Source: Crit Care. 2023 Nov 18;27:448. doi: 10.1186/s13054-023-04725-x (PMC10656773; doi:10.1186/s13054-023-04725-x)
Supplement: Supplementary file 1 — Additional file 1. Acute Injury Severity codes used to identify complete traumatic spinal cord injury patients within the Trauma Quality Improvement Program. [file 13054_2023_4725_MOESM1_ESM.docx]

**Additional file 1. Acute Injury Severity codes used to identify complete traumatic spinal cord injury patients within the Trauma Quality Improvement Program.**

| **Acute Injury Severity codes for Complete Spinal Cord Syndrome ^a^** | |
| --- | --- |
| Cervical Spine | 640220.5, 640221.5, 640222.5, 640224.5, 640226.5, 640228.5, 640229.6, 640230.6, 640232.6, 640234.6, 640236.6, 640260.5, 640261.5, 640262.5, 640264.5, 640266.5, 640268.5, 640269.6, 640270.6, 640272.6, 640274.6, 640276.6 |
| ^a^ Quadriplegia or paraplegia with no sensation | |
