# Supplementary material for: Factors influencing withdrawal of life-supporting treatment in cervical spinal cord injury: a large multicenter observational cohort study
Source: Crit Care. 2023 Nov 18;27:448. doi: 10.1186/s13054-023-04725-x (PMC10656773; doi:10.1186/s13054-023-04725-x)
Supplement: Supplementary file 2 — Additional file 2. Modelling Age as non-linear covariate. [file 13054_2023_4725_MOESM2_ESM.docx]

**Additional file 2.** **Modelling Age as non-linear covariate.**

In our multilevel logistic regression model, age was modelled as a non-linear continuous covariate in reference to regression modelling methodology described by Harrell.[1] Specifically, we fit non-hierarchical logistic regression models and compared models with age included as a linear or non-linear term with splines. We noted 1) a significant association between the non-linear terms for age and the log-odds of withdrawal of life-supporting treatment (WLST) from an analysis of variance of predictor terms (see Additional file 3); 2) a significant result in the likelihood ratio test comparing a model with age modelled as a linear covariate compared to a model with age modelled as a non-linear covariate (see Additional file 3); 3) a trend in the partial residual plots of a model with age modelled as a linear covariate (see Additional file 4); and 4) a non-linear relationship noted on inspection of a plot of the adjusted log-odds of WLST as a function of age (see Additional file 5).

**References**

1. Harrell FE. Regression Modeling Strategies [Internet]. Cham: Springer International Publishing; 2015 [cited 2023 Mar 29]. Available from: https://link.springer.com/10.1007/978-3-319-19425-7
