# Supplementary material for: Factors influencing withdrawal of life-supporting treatment in cervical spinal cord injury: a large multicenter observational cohort study
Source: Crit Care. 2023 Nov 18;27:448. doi: 10.1186/s13054-023-04725-x (PMC10656773; doi:10.1186/s13054-023-04725-x)
Supplement: Supplementary file 3 — Additional file 3. Results from analysis comparing a logistic regression model for age as a linear term, and non-linear terms. The table demonstrates the results from an analysis of variance of model terms from a non-linear model fit with age fit with restricted cubic splines with 4 knots. The likelihood ratio testa compares this model, with a nested model not including the non-linear terms. Abbreviations: GCS, Glasgow Coma Scale; AIS, Abbreviated Injury Scale [file 13054_2023_4725_MOESM3_ESM.docx]

**Additional file 3. Results from analysis comparing a logistic regression model for age as a linear term, and non-linear terms.** The table demonstrates the results from an analysis of variance of model terms from a non-linear model fit with age fit with restricted cubic splines with 4 knots. The likelihood ratio test^a^ compares this model, with a nested model not including the non-linear terms.

| **Predictor** | **P-Value** |
| --- | --- |
| Age – Composite Predictor | <0.001 |
| Age – Non-linear terms | 0.028 |
| Sex | 0.005 |
| Race | <0.001 |
| Insurance Type | 0.002 |
| Functionally Dependent | 0.104 |
| History of Stroke | 0.865 |
| History of Dementia | 0.023 |
| History of Disseminated Cancer | 0.971 |
| History of Chronic Renal Failure | 0.858 |
| Presenting GCS | <0.001 |
| Shock | 0.173 |
| Pre-Hospital Cardiac Arrest | <0.001 |
| Mechanism of injury | 0.001 |
| Head AIS ≥ 3 | 0.000 |
| Face AIS ≥ 3 | 0.823 |
| Neck AIS ≥ 3 | 0.064 |
| Thorax AIS ≥ 3 | 0.017 |
| Abdomen ≥ 3 | 0.992 |
| Spine Level of Injury | <0.001 |
| Upper Extremity AIS ≥ 3 | 0.727 |
| Lower Extremity AIS ≥ 3 | 0.068 |
| Hospital Bed-size | 0.044 |
| Hospital Teaching Status | 0.286 |
| Year of Injury | 0.333 |

^a^Likelihood Ratio test: χ^2^ = 10.32, p = .006

Abbreviations: GCS, Glasgow Coma Scale; AIS, Abbreviated Injury Scale.
