# Supplementary material for: Factors influencing withdrawal of life-supporting treatment in cervical spinal cord injury: a large multicenter observational cohort study
Source: Crit Care. 2023 Nov 18;27:448. doi: 10.1186/s13054-023-04725-x (PMC10656773; doi:10.1186/s13054-023-04725-x)
Supplement: Supplementary file 4 — Additional file 4. Partial residual plots of a logistic regression model fit with age as a linear predictor demonstrates a residual trend. [file 13054_2023_4725_MOESM4_ESM.docx]

**Additional file 4. Partial residual plots of a logistic regression model fit with age as a linear predictor demonstrates a residual trend.**
