# Supplementary material for: Factors influencing withdrawal of life-supporting treatment in cervical spinal cord injury: a large multicenter observational cohort study
Source: Crit Care. 2023 Nov 18;27:448. doi: 10.1186/s13054-023-04725-x (PMC10656773; doi:10.1186/s13054-023-04725-x)
Supplement: Supplementary file 5 — Additional file 5. Results from a full model with all covariates with age fit using restricted cubic splines demonstrates a non-linear relationship between the log-odds of withdrawal of life-supporting testament and age. Plots are adjusted to: Male sex; white race; private insurance; absence of functionally dependent status, prior stroke, dementia, disseminated cancer, chronic renal failure, shock, pre-hospital cardiac arrest; Glasgow Coma Scale score 15; Blunt Traumas; Abbreviated Injury Scale 1-2 score for head, face, neck, thorax, abdomen, upper extremity, and lower extremity; C4 and below spine injury; hospital bed size ≥ 600, university hospitals, injury year 2020. The grey shaded region represents a 95% confidence interval. Abbreviations: WLST, withdrawal of life-supporting treatment. [file 13054_2023_4725_MOESM5_ESM.docx]

**Additional file 5.** **Results from a full model with all covariates with age fit using restricted cubic splines demonstrates a non-linear relationship between the log-odds of withdrawal of life-supporting testament and age.**


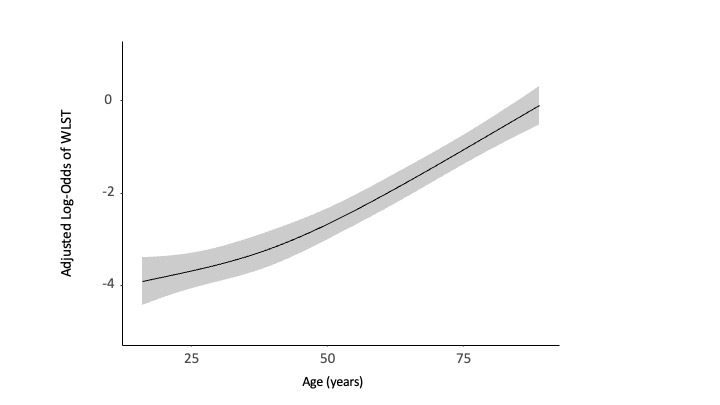


Additional file 5. Plots are adjusted to: Male sex; white race; private insurance; absence of functionally dependent status, prior stroke, dementia, disseminated cancer, chronic renal failure, shock, pre-hospital cardiac arrest; Glasgow Coma Scale score 15; Blunt Traumas; Abbreviated Injury Scale 1-2 score for head, face, neck, thorax, abdomen, upper extremity, and lower extremity; C4 and below spine injury; hospital bed size ≥ 600, university hospitals, injury year 2020. The grey shaded region represents a 95% confidence interval. Abbreviations: WLST, withdrawal of life supporting treatment.
