# Supplementary material for: Factors influencing withdrawal of life-supporting treatment in cervical spinal cord injury: a large multicenter observational cohort study
Source: Crit Care. 2023 Nov 18;27:448. doi: 10.1186/s13054-023-04725-x (PMC10656773; doi:10.1186/s13054-023-04725-x)
Supplement: Supplementary file 6 — Additional file 6. Results from sensitivity analysis excluding patients with presenting Glasgow Coma Scale between 3-8. The table provides estimated odds ratios for fixed effect predictors with a multilevel logistic regression model for withdrawal of life-supporting treatment. Abbreviations: CI, confidence interval; AIS, Abbreviated Injury Scale. [file 13054_2023_4725_MOESM6_ESM.docx]

**Additional file 6. Results from sensitivity analysis excluding patients with presenting Glasgow Coma Scale between 3-8.** The table provides estimated odds ratios for fixed effect predictors with a multi-level logistic regression model for withdrawal of life-supporting treatment.

| **Predictors** | **Odds Ratios** | ***95% CI*** | ***p-Value*** |
| --- | --- | --- | --- |
| Patient Level Characteristics | | | |
| **Age (years)** | - | - | <0.001^a,b^ |
| **Sex - Reference: Female** |  |  |  |
| Male | 1.47 | 1.09 – 1.98 | 0.011^a^ |
| **Race - Reference: White** |  |  |  |
| Black | 0.31 | 0.22 – 0.45 | <0.001^a^ |
| Asian | 0.52 | 0.26 – 1.02 | 0.056 |
| Other | 0.51 | 0.31 – 0.84 | 0.009^a^ |
| **Insurance Type – Reference: Private/Commercial** |  |  |  |
| Medicaid | 0.99 | 0.64 – 1.54 | 0.981 |
| Medicare | 1.36 | 0.99 – 1.88 | 0.057 |
| Self-Pay | 2.13 | 1.32 – 3.44 | 0.002^a^ |
| Other | 1.31 | 0.77 – 2.22 | 0.321 |
| **Comorbidities** |  |  |  |
| Functionally Dependent | 1.64 | 1.06 – 2.56 | 0.028^a^ |
| Prior Stroke | 1.03 | 0.54 – 1.97 | 0.929 |
| Dementia | 1.72 | 0.95 – 3.10 | 0.074 |
| Disseminated Cancer | 0.95 | 0.35 – 2.60 | 0.922 |
| Chronic Renal Failure | 1.11 | 0.49 – 2.53 | 0.797 |
| **Presenting Glasgow Coma Scale – Reference: 15** |  |  |  |
| 13-14 | 1.51 | 1.12 – 2.02 | 0.006^a^ |
| 9-12 | 1.42 | 1.00 – 2.01 | 0.048^a^ |
| **Shock** | 1.07 | 0.61 – 1.89 | 0.812 |
| **Pre-Hospital Cardiac Arrest** | 1.09 | 0.48 – 2.49 | 0.832 |
| **Mechanism of injury - Reference: Blunt** |  |  |  |
| Penetrating | 1.83 | 0.88 – 3.83 | 0.107 |
| **Spinal Cord Level of Injury – Reference: C4 and Below** |  |  |  |
| C3 and Above | 1.66 | 1.24 – 2.23 | 0.001^a^ |
| Severe Non-Spinal Injury – Reference: Body System AIS < 3 | | | |
| Head AIS ≥ 3 | 1.04 | 0.72 – 1.50 | 0.824 |
| Face AIS ≥ 3 | 0.54 | 0.05 – 5.30 | 0.593 |
| Neck AIS ≥ 3 | 1.28 | 0.88 – 1.87 | 0.199 |
| Thorax AIS ≥ 3 | 1.63 | 1.14 – 2.33 | 0.007^a^ |
| Abdomen AIS ≥ 3 | 1.47 | 0.60 – 3.62 | 0.402 |
| Upper Extremity AIS ≥ 3 | 1.75 | 0.35 – 8.82 | 0.500 |
| Lower Extremity AIS ≥ 3 | 1.48 | 0.76 – 2.89 | 0.244 |
| Hospital Level Characteristics | | | |
| **Hospital Size (Beds) – Reference: ≤200** |  |  |  |
| 201-400 | 0.63 | 0.34 – 1.15 | 0.134 |
| 401-600 | 0.87 | 0.47 – 1.58 | 0.641 |
| >600 | 0.64 | 0.35 – 1.17 | 0.148 |
| **Hospital Teaching Status – Reference: University** | | | |
| Non-teaching | 0.74 | 0.48 – 1.14 | 0.169 |
| Community | 0.88 | 0.67 – 1.17 | 0.382 |
| **Year of Injury – Reference: 2017** | | | |
| 2018 | 0.92 | 0.67 – 1.28 | 0.631 |
| 2019 | 1.11 | 0.81 – 1.52 | 0.514 |
| 2020 | 0.81 | 0.59 – 1.12 | 0.205 |

^a^Statistically significant

^b^Likelihood ratio test

Abbreviations: CI, confidence interval; AIS, Abbreviated Injury Scale.
