# Supplementary material for: Factors influencing withdrawal of life-supporting treatment in cervical spinal cord injury: a large multicenter observational cohort study
Source: Crit Care. 2023 Nov 18;27:448. doi: 10.1186/s13054-023-04725-x (PMC10656773; doi:10.1186/s13054-023-04725-x)
Supplement: Supplementary file 7 — Additional file 7. Analysis of missing data demonstrates that covariates are not missing completely at random based on Little’s Testa. Proportions of missing data by covariate and separated by complete cases and those with at least one element of missing data. Absolute standardized differences are tabulated. An absolute standardized difference >0.1 was prespecified to represent a meaningful difference between the group of patients with missing data and the group with complete cases. On inspection, there is no systematic trend in missing variables, suggesting they are likely missing at random. Abbreviations: GCS, Glasgow Coma Scale; AIS, Abbreviated Injury Scale. [file 13054_2023_4725_MOESM7_ESM.docx]

**Additional file 7. Analysis of missing data demonstrates that covariates are not missing completely at random based on Little’s Test^a^.** Proportions of missing data by covariate and separated by complete cases and those with at least one element of missing data. Absolute standardized differences are tabulated. An absolute standardized difference >0.1 was prespecified to represent a meaningful difference between the group of patients with missing data and the group with complete cases. On inspection, there is no systematic trend in missing variables, suggesting they are likely missing at random.

|  | **Cohort Before Excluding Cases with Missing Data N = 5582** | | **Complete Cases N = 5070 (90.83%)** | **Cases with Missing Data N= 512 (9.17 %)** | **Absolute Standardized Difference** |
| --- | --- | --- | --- | --- | --- |
| **Age** |  | |  |  |  |
| Mean (SD) | 49.4 (20.1) | | 49.6 (20.1) | 47.5 (20.2) | 0.104^b^ |
| Median [Min, Max] | 51.0 [16.0, 89.0] | | 52.0 [16.0, 89.0] | 46.0 [16.0, 89.0] | - |
| Missing | 0 (0%) | | 0 (0%) | 0 (0%) | - |
| **Sex** |  | |  |  | 0.013 |
| Female | 1109 (19.9%) | | 1005 (19.8%) | 104 (20.3%) | - |
| Male | 4472 (80.1%) | | 4065 (80.2%) | 407 (79.5%) | - |
| Missing | 1 (0.0%) | | 0 (0%) | 1 (0.2%) | - |
| **Race** |  | |  |  | 0.135^b^ |
| White | 3449 (61.8%) | | 3228 (63.7%) | 221 (43.2%) | - |
| Black | 1361 (24.4%) | | 1267 (25.0%) | 94 (18.4%) | - |
| Asian | 154 (2.8%) | | 135 (2.7%) | 19 (3.7%) | - |
| Other | 471 (8.4%) | | 440 (8.7%) | 31 (6.1%) | - |
| Missing | 147 (2.6%) | | 0 (0%) | 147 (28.7%) | - |
| **Insurance Type** |  | |  |  | 0.109^b^ |
| Private/Commercial | 2145 (38.4%) | | 2001 (39.5%) | 144 (28.1%) | - |
| Medicaid | 1183 (21.2%) | | 1090 (21.5%) | 93 (18.2%) | - |
| Medicare | 1241 (22.2%) | | 1154 (22.8%) | 87 (17.0%) | - |
| Self-Pay | 565 (10.1%) | | 519 (10.2%) | 46 (9.0%) | - |
| Other | 338 (6.1%) | | 306 (6.0%) | 32 (6.3%) | - |
| Missing | 110 (2.0%) | | 0 (0%) | 110 (21.5%) | - |
| **Functionally Dependent** |  | |  |  | 0.038 |
| No | 5380 (96.4%) | | 4890 (96.4%) | 490 (95.7%) | - |
| Yes | 202 (3.6%) | | 180 (3.6%) | 22 (4.3%) | - |
| Missing | 0 (0%) | | 0 (0%) | 0 (0%) | - |
| **Stroke** |  | |  |  | 0.022 |
| No | 5488 (98.3%) | | 4986 (98.3%) | 502 (98.0%) | - |
| Yes | 94 (1.7%) | | 84 (1.7%) | 10 (2.0%) | - |
| Missing | 0 (0%) | | 0 (0%) | 0 (0%) | - |
| **Dementia** |  | |  |  | 0.048 |
| No | 5485 (98.3%) | | 4985 (98.3%) | 500 (97.7%) | - |
| Yes | 97 (1.7%) | | 85 (1.7%) | 12 (2.3%) | - |
| Missing | 0 (0%) | | 0 (0%) | 0 (0%) | - |
| **Disseminated Cancer** |  | |  |  | 0.036 |
| No | 5547 (99.4%) | | 5037 (99.3%) | 510 (99.6%) | - |
| Yes | 35 (0.6%) | | 33 (0.7%) | 2 (0.4%) | - |
| Missing | 0 (0%) | | 0 (0%) | 0 (0%) | - |
| **Chronic Renal Failure** |  | |  |  | 0.024 |
| No | 5526 (99.0%) | | 5019 (99.0%) | 507 (99.0%) | - |
| Yes | 55 (1.0%) | | 51 (1.0%) | 4 (0.8%) | - |
| Missing | 1 (0.0%) | | 0 (0%) | 1 (0.2%) | - |
| **Presenting GCS** |  | |  |  | 0.064 |
| 3-8 | 1604 (28.7%) | | 1487 (29.3%) | 117 (22.9%) | - |
| 9-12 | 464 (8.3%) | | 428 (8.4%) | 36 (7.0%) | - |
| 13-14 | 702 (12.6%) | | 651 (12.8%) | 51 (10.0%) | - |
| 15 | 2680 (48.0%) | | 2504 (49.4%) | 176 (34.4%) | - |
| Missing | 132 (2.4%) | | 0 (0%) | 132 (25.8%) | - |
| **Shock** |  | |  |  | 0.014 |
| No | 5260 (94.2%) | | 4776 (94.2%) | 484 (94.5%) | - |
| Yes | 322 (5.8%) | | 294 (5.8%) | 28 (5.5%) | - |
| Missing | 0 (0%) | | 0 (0%) | 0 (0%) | - |
| **Pre-Hospital Cardiac Arrest** | |  |  |  | 0.018 |
| No | 4894 (87.7%) | | 4508 (88.9%) | 386 (75.4%) | - |
| Yes | 613 (11.0%) | | 562 (11.1%) | 51 (10.0%) | - |
| Missing | 75 (1.3%) | | 0 (0%) | 75 (14.6%) | - |
| **Mechanism of injury** |  | |  |  | 0.042 |
| Blunt | 5039 (90.3%) | | 4590 (90.5%) | 449 (87.7%) | - |
| Penetrating | 534 (9.6%) | | 480 (9.5%) | 54 (10.5%) | - |
| Missing | 9 (0.2%) | | 0 (0%) | 9 (1.8%) | - |
| **Head AIS** |  | |  |  | 0.008 |
| 1-2 | 4475 (80.2%) | | 4066 (80.2%) | 409 (79.9%) | - |
| 3+ | 1107 (19.8%) | | 1004 (19.8%) | 103 (20.1%) | - |
| Missing | 0 (0%) | | 0 (0%) | 0 (0%) | - |
| **Face AIS** |  | |  |  | 0.031 |
| 1-2 | 5549 (99.4%) | | 5039 (99.4%) | 510 (99.6%) |  |
| 3+ | 33 (0.6%) | | 31 (0.6%) | 2 (0.4%) | - |
| Missing | 0 (0%) | | 0 (0%) | 0 (0%) | - |
| **Neck AIS** |  | |  |  | 0.038 |
| 1-2 | 4875 (87.3%) | | 4422 (87.2%) | 453 (88.5%) | - |
| 3+ | 707 (12.7%) | | 648 (12.8%) | 59 (11.5%) | - |
| Missing | 0 (0%) | | 0 (0%) | 0 (0%) | - |
| **Thorax AIS** |  | |  |  | 0.012 |
| 1-2 | 4494 (80.5%) | | 4084 (80.6%) | 410 (80.1%) | - |
| 3+ | 1088 (19.5%) | | 986 (19.4%) | 102 (19.9%) | - |
| Missing | 0 (0%) | | 0 (0%) | 0 (0%) | - |
| **Abdomen AIS** |  | |  |  | 0.033 |
| 1-2 | 5375 (96.3%) | | 4885 (96.4%) | 490 (95.7%) | - |
| 3+ | 207 (3.7%) | | 185 (3.6%) | 22 (4.3%) | - |
| Missing | 0 (0%) | | 0 (0%) | 0 (0%) | - |
| **Spine Cord Level of Injury** |  | |  |  | 0.015 |
| C4 and Below | 4327 (77.5%) | | 3933 (77.6%) | 394 (77.0%) | - |
| C3 and Above | 1255 (22.5%) | | 1137 (22.4%) | 118 (23.0%) | - |
| Missing | 0 (0%) | | 0 (0%) | 0 (0%) | - |
| **Upper Extremities** |  | |  |  | 0.129^b^ |
| 1-2 | 5540 (99.2%) | | 5028 (99.2%) | 512 (100%) | - |
| 3+ | 42 (0.8%) | | 42 (0.8%) | 0 (0%) | - |
| Missing | 0 (0%) | | 0 (0%) | 0 (0%) | - |
| **Lower Extremities** |  | |  |  | 0.049 |
| 1-2 | 5289 (94.8%) | | 4799 (94.7%) | 490 (95.7%) | - |
| 3+ | 293 (5.2%) | | 271 (5.3%) | 22 (4.3%) | - |
| Missing | 0 (0%) | | 0 (0%) | 0 (0%) | - |
| **Hospital Beds** |  | |  |  | 0.102^b^ |
| <= 200 | 221 (4.0%) | | 202 (4.0%) | 19 (3.7%) | - |
| 201-400 | 1320 (23.6%) | | 1214 (23.9%) | 106 (20.7%) | - |
| 401-600 | 1704 (30.5%) | | 1553 (30.6%) | 151 (29.5%) | - |
| > 600 | 2337 (41.9%) | | 2101 (41.4%) | 236 (46.1%) | - |
| Missing | 0 (0%) | | 0 (0%) | 0 (0%) | - |
| **Hospital Teaching Status** |  | |  |  | 0.090 |
| university | 3104 (55.6%) | | 2835 (55.9%) | 269 (52.5%) | - |
| nonteaching | 570 (10.2%) | | 521 (10.3%) | 49 (9.6%) | - |
| community | 1848 (33.1%) | | 1714 (33.8%) | 134 (26.2%) | - |
| Missing | 60 (1.1%) | | 0 (0%) | 60 (11.7%) | - |
| **Year of Injury** |  | |  |  | 0.081 |
| 2017 | 1366 (24.5%) | | 1255 (24.8%) | 111 (21.7%) | - |
| 2018 | 1373 (24.6%) | | 1249 (24.6%) | 124 (24.2%) | - |
| 2019 | 1364 (24.4%) | | 1230 (24.3%) | 134 (26.2%) | - |
| 2020 | 1479 (26.5%) | | 1336 (26.4%) | 143 (27.9%) | - |
| Missing | 0 (0%) | | 0 (0%) | 0 (0%) | - |

^a^Little’s Test χ^2^ = 6173.77, degrees of freedom 407, p < .001

^b^Absolute standardized difference corresponds to meaningful difference (>0.1)

Abbreviations: GCS, Glasgow Coma Scale; AIS, Abbreviated Injury Scale.
