# Supplementary material for: Factors influencing withdrawal of life-supporting treatment in cervical spinal cord injury: a large multicenter observational cohort study
Source: Crit Care. 2023 Nov 18;27:448. doi: 10.1186/s13054-023-04725-x (PMC10656773; doi:10.1186/s13054-023-04725-x)
Supplement: Supplementary file 8 — Additional file 8. Percentage of missing data by variable demonstrates that less than 5% data are missing per covariate. Abbreviations: GCS, Glasgow Coma Scale; AIS, Abbreviated Injury Scale. [file 13054_2023_4725_MOESM8_ESM.docx]

**Additional file 8.** **Percentage of missing data by variable demonstrates that less than 5% data are missing per covariate.**

Abbreviations: GCS, Glasgow Coma Scale; AIS, Abbreviated Injury Scale.
